# Immunoprotective effect of an in silico designed multiepitope cancer vaccine with BORIS cancer-testis antigen target in a murine mammary carcinoma model

**DOI:** 10.1038/s41598-021-01770-w

**Published:** 2021-11-30

**Authors:** Elham Mahdevar, Amirhosein Kefayat, Ashkan Safavi, Amirhossein Behnia, Seyed Hossein Hejazi, Amaneh Javid, Fatemeh Ghahremani

**Affiliations:** 1 Department of Biological Sciences, Faculty of Science and Engineering, Science and Arts University, Yazd, Iran; 2 Department of Oncology, Isfahan University of Medical Sciences, Isfahan, Iran; 3 Department of Biology, Science and Research Branch, Islamic Azad University, Tehran, Iran; 4 Department of Biology, Faculty of the Basic Sciences, Shahrekord Branch, Islamic Azad University, Shahrekord, Iran; 5 Department of Parasitology and Mycology, School of Medicine, Isfahan University of Medical Sciences, Isfahan, Iran; 6 Department of Medical Physics and Radiotherapy, School of Paramedicine, Arak University of Medical Sciences, Arak, Iran

**Keywords:** Breast cancer, Metastasis, Tumour immunology, Vaccines

## Abstract

In our previous study, immunoinformatic tools were used to design a novel multiepitope cancer vaccine based on the most immunodominant regions of BORIS cancer-testis antigen. The final vaccine construct was an immunogenic, non-allergenic, and stable protein consisted of multiple cytotoxic T lymphocytes epitopes, IFN-γ inducing epitopes, and B cell epitopes according to bioinformatic analyzes. Herein, the DNA sequence of the final vaccine construct was placed into the pcDNA3.1 vector as a DNA vaccine (pcDNA3.1-VAC). Also, the recombinant multiepitope peptide vaccine (MPV) was produced by a transfected BL21 *E. coli* strain using a recombinant pET-28a vector and then, purified and screened by Fast protein liquid chromatography technique (FPLC) and Western blot, respectively. The anti-tumor effects of prophylactic co-immunization with these DNA and protein cancer vaccines were evaluated in the metastatic non-immunogenic 4T1 mammary carcinoma in BALB/c mice. Co-immunization with the pcDNA3.1-VAC and MPV significantly (*P* < 0.001) increased the serum levels of the MPV-specific IgG total, IgG2a, and IgG1. The splenocytes of co-immunized mice exhibited a significantly higher efficacy to produce interleukin-4 and interferon-γ and proliferation in response to MPV in comparison with the control. The prophylactic co-immunization regime caused significant breast tumors’ growth inhibition, tumors’ weight decrease, inhibition of metastasis formation, and enlarging tumor-bearing mice survival time, without any considerable side effects. Taking together, this cancer vaccine can evoke strong immune response against breast tumor and inhibits its growth and metastasis.

## Introduction

Breast cancer is the most common neoplasm among women, accounting for one-third of newly diagnosed malignancies^1^. Although current therapeutic approaches use radiotherapy, chemotherapy, and surgery for the treatment of breast cancer, the patient’s prognosis isn’t still satisfying, especially in metastatic patients^2^. Also, the side effects of these therapeutic approaches have limited their efficacy. Therefore, immunotherapy and especially cancer vaccines have gained lots of attention for breast cancer treatment due to tumor specificity, good patients’ tolerance, safety, and long-term immune memory which can prevent tumor recurrence and relapse^3^.

Selecting an appropriate target antigen is deeply determinative in the efficacy of the designed anticancer vaccine^4^. Cancer-testis antigens (CTAs) are tumor-associated antigens whose expressions are limited to tumors and germ cells of the testis (sometimes female reproductive organs and trophoblasts), but not in somatic normal tissues^5^. Their overexpression can be detected in tumors with different origins and its level is highly associated with the tumor stage, tumor invasion, and patient’s prognosis. Besides, CTAs have exhibited high tumor-specificity and strong immunogenicity. Taking together, CTAs may constitute potentially promising targets for designing anticancer vaccines^6^. BORIS (Brother of Regulator of Imprinted Sites) is a CTA with high expression in different malignancies^7–9^. Although BORIS antigen is overexpressed by various breast cancer cell lines, it can’t be detected in the normal mammary cell lines and healthy breast tissues^10^. Breast cancer cells’ viability is deeply associated with BORIS expression, as silencing of BORIS using siRNA reduces their viability^8^. Besides, BORIS's role in tumor progression was demonstrated through activation of the progesterone and estrogen receptors’ genes promoters^11^. Moreover, its expression is related to rising stemness traits and epithelial-to-mesenchymal transition (EMT) in breast cancer cells. BORIS is used as a target biomarker of cancer stem cells which are the key role players in tumor recurrence, distant organs metastasis formation, and treatment resistance^12,13^. Besides, significantly higher levels of cell-free BORIS mRNA were detected in the serum of patients with malignant breast carcinoma compared with patients with benign breast lesions or healthy women^14^. In general, these findings demonstrate that BORIS can be an ideal target for breast cancer patients’ vaccination.

Although antigen selection plays a key role in designing cancer vaccines, the use of whole protein can decrease vaccine efficiency^15^. Previous studies have demonstrated that immunodominant epitopes of antigens can be more effective than the whole protein vaccination^16^. In our previous study^17^, the most immunodominant regions of BORIS antigen were identified by immunoinformatic approaches. The selected regions were linked together with appropriate linkers and underwent multiple modifications to form the final DNA and peptide cancer vaccines’ construct. The final vaccine construct was an immunogenic, non-allergenic, and stable protein that contained numerous cytotoxic T lymphocytes (CTL) epitopes, IFN-γ inducing epitopes, and several linear and conformational B cell epitopes according to bioinformatic analyzes. The main purpose of the present study is in vivo assessment of this in silico designed cancer vaccine efficacy in different aspects, in a syngeneic non-immunogenic metastatic murine breast cancer model. Biotechnological methods were employed to produce and purify a considerable amount of the designed multiepitope DNA and peptide vaccines for in vivo experiments. Subsequently, the designed multiepitope peptide and DNA vaccines' ability for immune response evoking and inducing anti-tumor effects were investigated by measuring antibody production, cytokine secretion, 4T1 breast tumor growth progression, metastasis, and tumor-bearing mice survival time.

## Materials and methods

### Study design

**Figure 1:**
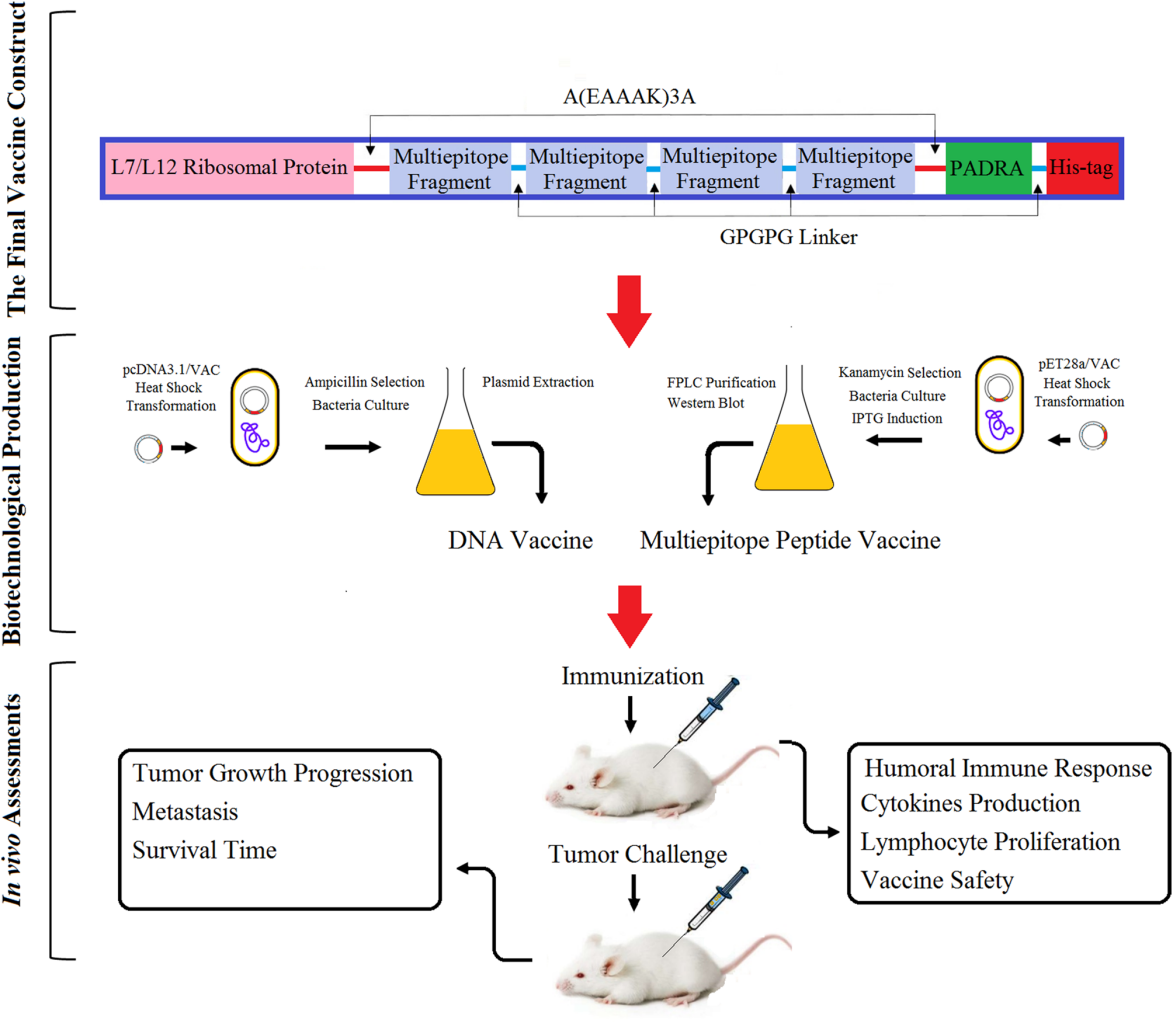
Schematic illustration of the multiepitope peptide vaccine construct and study steps including multiepitope peptide and DNA vaccines production, purification, and in vivo assessment of its immunoprotective effects in a non-immunogenic metastatic murine breast cancer model.

Based on our previous study^18^, the 265–312, 320–400, 416–478, and 498–533 residues of BORIS antigen were predicted as the most immunodominant fragments which contained multiple MHCs, CTL, CD4 + T-helper, and B cell epitopes for both mouse and human according to different immunoinformatic tools. The selected fragments were linked together by GPGPG linkers. Also, a universal T helper epitope (PADRE) and the L7/L12 ribosomal protein of mycobacterium as a TLR-4/MD-2 agonist were incorporated in the construct by A(EAAAK)3A linker to form the final vaccine construct. The amino acid sequence of the final vaccine construct is illustrated in Fig. S1. The final vaccine construct was reverse translated and codon-optimized. The DNA sequence of the final construct was ordered to the GeneCust Company (Dudelange, Luxemburg) in pcDNA3.1 expression vector as a DNA vaccine and also, in pET-28a vector for multiepitope peptide vaccine production in the prokaryote host. Then, the pcDNA3.1 and pET-28a were cloned in the *E. coli* host. The recombinant vectors of the multiepitope peptide vaccine (MPV) were expressed, produced, and the multiepitope peptide vaccine was purified by the FPLC method. Subsequently, the immune response, anti-tumor effects, and safety of the designed multiepitope peptide and DNA vaccines were investigated by assessing antibody production, cytokines production, lymphocytes proliferation, vital organs histopathological exams, 4T1 breast tumor growth progression, metastasis, and tumor-bearing mice survival time. All the study steps are schematically illustrated in Fig. 1.

### Cloning of the recombinant pET-28a (pET-28a-VAC) and pcDNA3.1 (pcDNA3.1-VAC) in *E. coli*

After vectors preparation, the pcDNA3.1-VAC and pET-28a-VAC vectors **(**GeneCust, Luxembourg) were distinctly incubated with the competent *E. coli* Top10 strain. The bacteria were transfected by the heat shock method and left for 60 min in Luria–Bertani (LB) broth culture media (Sigma, USA) at 37 C˚. 100 μL of each culture media containing the transfected bacteria (pcDNA3.1 or pET-28a) were plated onto LB agar plates containing Ampicillin (100 μg/mL) (Sigma, USA), Tetracycline (10 μg/mL) (Sigma, USA) for pcDNA3.1-VAC transfected bacteria and Kanamycin (50 μg/mL) (Sigma, USA) for pET-28a-VAC transfected bacteria. They were cultured for 16 h at 37 C˚ and the positive colonies were confirmed by digestion and colony PCR. The utilized forward and reverse primers were GCAGAAACATGGCGAAAACG and TTCATGAAACACGGCAGAGC as forward and reverse primers, respectively. In addition, the plasmids were extracted from confirmed colonies by miniperp kit (QIAGEN, USA) and then digested by Nco1 (Invitrogen, USA) and Xho1 (Invitrogen, USA) restriction enzymes. The digested plasmids were screened by agarose gel electrophorese to confirm the recombinant colonies. Cellular pellets were collected by centrifugation and the extracted pcDNA3.1-VAC was collected as the DNA vaccine. Besides, the pET-28a-VAC plasmid was purified and used for the transformation of the *E. coli* BL21 (DE3) strain. All of these protocols were done according to our previous study^19^.

### The recombinant multiepitope vaccine expression and production

The L929 cells (murine fibroblast) were purchased from the Pastor Institute of Tehran, Iran. The cells were seeded at a density of 5 × 10^5^ cells per well, fulfilled by RPMI containing 10% fetal bovine serum (FBS). To confirm the pcDNA3.1-VAC expression and production in the eukaryote host, it was transfected to L929 cells by Turbofect (Thermo Scientific, USA) according to the manufacturer’s recommendations. 8 h after transfection, culture media was replaced and incubated for another 48 h. Then, the recombinant multiepitope peptide vaccine expression was detected and confirmed by immunoblotting. In addition, a single transformed colony was cultured into LB broth, containing Kanamycin (50 μg/mL) (Sigma, USA). After reaching the 0.6 optical density (OD) at 600 nm. 15 mL of the culture media was removed as an uninduced control and the remainder was mixed with IPTG (Sigma, USA) at the final concentration of 1 mM and incubated for 3 more hours. The bacteria cells were harvested by centrifugation at 5000 g for 5 min at 4 C˚ and the pellets were lysate by sonication in PBS buffer supplied with 1% protease inhibitor (Roche, Germany) for western blot analysis. All of these protocols were done according to our previous study^19^.

### Immunoblotting of the recombinant multiepitope peptide vaccine

For western blot analysis, hosts protein solutions were obtained by boiling in a sample buffer that contained Tris–HCl (pH = 6.8, 0.5 M) (Sigma, USA), SDS (10% W/V) (Sigma, USA), Glycerol (50% V/V) (Sigma, USA), and Bromo Phenol Blue (0.5% W/V) (Sigma, USA), for 5 min. 40 μL of each sample was loaded in SDS-PAGE (13%) (Sigma, USA) under non-reducing conditions to separate protein bonds. The protein constituents were electrophoretically blotted onto a polyvinylidene difluoride membrane (Millipore, USA). The membrane was blocked in 5% skim milk/0.1% Tween 20 in PBS (pH 7.4), and then the reactivity of the transferred protein(s) was evaluated with 1 μg/mL monoclonal Anti-poly Histidine − Peroxidase antibody. Finally, the detection was performed with tetramethylbenzidine substrate (Sigma, USA). All of these protocols were done according to our previous study^19^.

### Harvesting and lysis of the bacterial cells to increase production efficacy

The recombinant *E. coli* cells were separated from the culture media by centrifugation at 4,000 rpm for 20 min at 4 °C, and the supernatant was discarded. Subsequently, the falcon tubes which contained the cell pellets were located in a dry ice/ethanol bath for 4 min, transferred to cold water for 60 s to melt the ice along the walls of the tube, and a sterile plastic spatula was used to break the pellet free. The pellets were transferred and suspended in 200 mL of neutral (N)-lysis buffer (50 mM Tris–HCl, pH 7.5, containing 0.3 M NaCl and 0.5 mM EDTA) in a 500 mL flask. To digest the cell wall, lysozyme (0.5 mg/mL) was added to the buffer and incubated for 2 h at room temperature. The *E. coli* cells were lysed using 20 mL of 10X Mg/Mn salt solution (100 mM MgCl_2_ and 10 mM MnCl_2_). DNA was removed by DNase (10 μg/mL, 30 min at room temperature). The resulting *E. coli* lysate (~ 220 mL) was placed into a dialysis bag (10 kDa molecular weight cut-off) in 4 L of buffer (10 mM Tris–HCl, pH 7.4, containing 0.5 mM EDTA). The dialysis buffer was changed twice over a 16–24 h period. At the end of the dialysis, centrifugation at 12,000 rpm for 30 min at 10 °C was employed to remove the cell debris. All of these protocols were done according to our previous study^19^.

### Purification of the recombinant multiepitope peptide vaccine

The purification of the recombinant His-tagged peptide vaccine was optimized for maximal yield and purity by employing a DEAE Sepharose column, followed by nickel affinity chromatography. DEAE Sepharose ion exchange (HiPrep 16/10 QFF) was conducted using Fast Performance Liquid Chromatography (FPLC) (Bio-Rad, USA). The dialyzed and centrifuged recombinant bacteria lysis supernatant was applied to a DEAE-Sepharose Fast Flow column (GE Healthcare, Piscataway, NJ) that was equilibrated with dialysis buffer (10 mM Tris–HCl, pH 7.4, containing 0.5 mM EDTA). The peptide vaccine was eluted with an increasing salt gradient of up to 1 M NaCl in dialysis buffer. The peptide vaccine-containing fractions were pooled and dialyzed against 3 L of 20 mM Tris–HCl buffer (pH 7.5), containing 200 mM NaCl. The dialyzed solution was applied to a 1 mL Nickel Nitrilotriacetic acid (Ni–NTA) column and eluted by gravity flow after the column equilibrating with dialysis buffer. The purification of the peptide vaccine was based on the His-tag protocol. After the protein was loaded onto the Ni–NTA column, it was washed with 10-bed volumes of 20 mM Tris–HCl buffer (pH 7.5), containing 200 mM NaCl and up to 20 mM imidazole. 2 mL fractions of eluate were collected throughout the entire purification protocol. Recombinant peptide vaccine was eluted from the Ni–NTA column with dialysis buffer containing 200 mM imidazole. The different steps of purification (i.e. ion exchange and affinity chromatography) were monitored by SDS-PAGE, and the peptide vaccine presence was confirmed by immunoblotting. The total amount of the extracted recombinant peptide vaccine was determined using the Bradford method (Start Quick Assay Protein Bradford kit). All of these protocols were done according to our previous study^19^. The LPS level in the extracted recombinant peptide vaccine was determined by the Chromogenic LAL assay according to previous studies^20,21^.

### Animal care, handling, and ethics

All animal experiments complied with the ARRIVE guidelines and were carried out in accordance with the guidelines of the European Communities Council Directive (2010/63/UE). Also, all the procedures and steps were approved by the ACECR (Academic Center for Education, Culture and Research) Research Ethics Committee of Mashhad, Iran (IR.ACECR.JDM.REC.1399.002). Female BALB/c mice (weight: 22 ± 2 g) were purchased from the Pasteur Institute of Tehran, Iran. The animals had complete access to standard animal lab chow and water all over the study. The animals’ lab conditions were tuned as follows: 50 ± 10% relative humidity, 24 ± 2 ºC temperature, and 12 h light/12 h dark cycle condition. In this study, standardized humane endpoints based on the current guidelines for endpoints in animal tumor studies were used^22–24^. The animal scarifying was done by the overdose of ketamine/xylazine.

### Animal immunization

The animals were acclimated for at least two weeks before entering the study. Subsequently, the mice were randomly divided into six groups (n = 25). Each mouse in each group was injected four times with one-week interval. As Table 1 illustrates, the immunization regime and schedule for each group was as follow: (1st group: PBS) 100 μL PBS subcutaneously (s.c); (2nd group: IFA) s.c injection with 100 μL incomplete Freund’s adjuvant (IFA) which was formulated with PBS in 50% V/V; (3rd group: pcDNA3.1) intramuscular (in the hind leg of the mouse) injection with 100 µg pcDNA3.1 in 100 μL PBS; (4th group: pcDNA3.1-VAC) intramuscular (i.m) injection with 100 µg pcDNA3.1-VAC in 100 μL PBS; (5th group: multiepitope peptide vaccine (MPV)) s.c injection with a mixture of the MPV (100 μg in 50 μL of PBS) emulsified with 50 μL IFA 50% V/V; (6th group: pcDNA3.1-VAC/MPV) injection with 100 µg pcDNA in 100 µL PBS (i.m) for the first and second injections and a mixture of the MPV (100 μg in 50 μL PBS) emulsified with 50 μL IFA 50% V/V for the third and fourth ones.

**Table 1:** Vaccination schedule at different immunization groups.

Group	1st injection	2nd injection	3rd injection	4th injection
1st	PBS	PBS	PBS	PBS
2nd	IFA	IFA	IFA	IFA
3rd	pcDNA 3.1	pcDNA 3.1	pcDNA 3.1	pcDNA 3.1
4th	pcDNA 3.1-VAC	pcDNA 3.1-VAC	pcDNA 3.1-VAC	pcDNA 3.1-VAC
5th	MPV-IFA	MPV-IFA	MPV-IFA	MPV-IFA
6th	pcDNA 3.1-VAC	pcDNA 3.1-VAC	MPV-IFA	MPV-IFA

IFA: Incomplete Freund’s adjuvant, PBS: Phosphate buffer solution, n: Number of mice per group, pcDNA 3.1/VAC: The multiepitope DNA vaccine, MPV: The multiepitope peptide vaccine.

### Antibodies determination by enzyme-linked immunosorbent assay (ELISA)

Two weeks after the last injection of the immunization schedule, a fraction of immunized mice in each group (n = 5) were sacrificed by the overdose of ketamine/xylazine, and their blood was collected by cardiac puncture. An indirect ELISA assay kit (R&D Systems, USA) was employed to determine the MPV-specific serum levels of IgG total, IgG1, and IgG2a antibodies. In brief, 96-well microtiter plates were coated with the MPV in 50 mM carbonate buffer (pH 9.6) overnight at 4 °C. Then, the wells were washed three times with PBS supplied with 0.05% Tween 20. At the next step, the blocking solution (bovine serum albumin 1%) was added to the wells and the plate was incubated for 1 h at 37 °C. After three times wash with PBS, the sera were added (three wells for each sample) and the plate was incubated for another 1 h at 37 °C. HRP-conjugated goat anti-mouse IgG total, IgG1, or IgG2a (Abcam, USA) antibodies were employed as the secondary antibody. Finally, the orthophenylene diamine (Sigma, USA) and 0.15% H_2_O_2_ (Sigma, USA) were added and incubated for 30 min to visualize the immune complexes. After stopping the reaction by adding H_2_SO_4_, a microplate reader (Bio-Rad 680, USA) was employed to read the absorbance at 490 nm. Complete culture media was used as blank.

### Cytokines secretion assay

Two weeks after the last injection of the immunization schedule, five immunized mice from each group (n = 5) were sacrificed by overdose of ketamine/xylazine and their spleens were harvested in a completely aseptic condition. The splenocytes were cultured in 96-well cell culture plates. Then, the cultured splenocytes were re-stimulated with the MPV (10 mg/mL) and incubated in a cell culture incubator for 1 h. Subsequently, interleukin-4 (IL-4) and interferon-gamma (IFN-γ) cytokines levels were measured in the wells’ supernatant using commercial ELISA kits (Abcam, USA), following the manufacturer's instruction. Complete cell culture media was used as blank. Three wells for each sample were prepared.

### Antigen-specific proliferation assay

According to previous studies^25,26^, the cultured splenocytes were seeded (1.5 × 10^4^ cells/well) in a 96‑well plate. For each sample, at least three wells were prepared. Subsequently, the MPV (2 μg/well) was added to the wells as the recall antigen to stimulate the splenocytes. Some wells weren’t treated with the MPV to be used as negative controls. On the other hand, the phytohemagglutinin‑A (PHA) treated wells (5 μg/ml) were used as positive controls. MTT assay was used to measure lymphocyte proliferation according to the manufacturer’s recommendations. A microplate ELISA reader was used to measure the absorbance of each well at a 570 nm wavelength. All tests were performed in triplicate for each mouse, and results were expressed as stimulation index. The stimulation index (SI) was calculated using the following Eq. (1):

1 $$ {\text{Stimulation}}\,{\text{index}} \,\left( {{\text{SI}}} \right) = \frac{{\left( {{\text{Stimulated}}\,{\text{wells}}^{\prime}{\text{OD}} - {\text{Blank}} \,{\text{OD}}} \right)}}{{\left( { {\text{unstimulated}}\,{\text{wells}}^{\prime}{\text{OD}} - {\text{Blank}}\,{\text{OD}}} \right)}} $$

### Tumor challenge

4T1 murine mammary carcinoma cell line was purchased from the Pasteur Institute of Tehran, Iran. RPMI medium (Sigma, USA) supplied with 10% fetal bovine serum (FBS) (Sigma, USA) and 1% antibiotics mixture containing penicillin (Sigma, USA) and streptomycin (Sigma, USA) was used as the culture media. The cells were incubated at 37 °C in a humidified incubator in a 5% CO_2_ atmosphere. After reaching the appropriate confluence at the flasks’ bottom**,** the cells were detached by trypsin (Sigma, USA). 14 days after the last immunization, the cancer cells were subcutaneously inoculated. Each mouse was s.c injected (into the 4th left mammary gland fat pad) with 1 × 10^6^ 4T1 cells suspended in 50 µL of DMEM-F12. The injection site was shaved and sterilized before cancer cell injection. To determine tumors’ growth progression, the greatest longitudinal diameter (length) and the greatest transverse diameter (width) of the tumors were measured every 3 days for 27 days. Then, the tumor’s volume was calculated by the Eq. (2). The tumor inoculation day was determined as day 0 at the tumor growth progression chart. For survival analysis, the tumor-bearing mice (n = 8) were closely monitored for 80 days after tumor implantation. The animals’ death was recorded every day. The standardized humane endpoint used to euthanize animals was the failure to eat and drink for over 3 days and without any limb movement.

2 $$ {\text{Tumor}}\,{\text{volume}} = \frac{{\left( {{\text{Tumor}}\,{\text{length}}} \right) \times \left( {{\text{Tumor}}\,{\text{width}}} \right)^{2} }}{2} $$

On the 40th day after cancer cell implantation, 5 mice from each group were sacrificed by the overdose of ketamine/xylazine, and their livers and lungs were harvested and fixed in the 10% formalin neutral buffer solution. An automatic tissue processor (Sakura, Japan) was used to process the fixed samples. Then, a microtome (Leica Biosystems, Germany) was employed to cut 4 µm thickness serial sections from the paraffin-embedded blocks. The sections were stained with Hematoxylin & Eosin (H&E) staining protocol according to previous studies^27^. A minimum of 10 random microscopic fields were observed under 10 × objective lens of a light microscope (Olympus, Japan) to report the average number of metastatic colonies per microscopic field of the liver. However. All over the lungs were analyzed for metastatic colonies.

### Histopathological analysis

10 Female BALB/c mice were randomly divided into 2 groups (n = 5) including Control and pcDNA3.1-VAC/MPV. The mice in the 1st group didn’t receive any treatment. The 2nd group underwent the pcDNA3.1-VAC/MPV immunization regime, the same as mentioned in 2.10. Section (Table 1). The mice were under close observation for mortality, appearance, behavioral pattern changes such as weakness, aggressiveness, food or water refusal, and pain or any signs of illness for 30 days after the last injection. Also, the animals were weighed every day to monitor their body weight. On the 30th day, the mice were sacrificed by overdose of ketamine/xylazine, and their vital organs including heart, liver, brain, and lungs were harvested and fixed in 10% formalin neutral buffer solution. After preparing the H&E stained slides, the histopathological analyzes were done by employing a light microscope (Olympus, Japan) and histological photographs were captured by a digital light microscope (Olympus, Japan).

### Statistical analysis

JMP 11.0 (version 11.0, SAS Institute, Japan) software (https://www.jmp.com/) was used for statistical analyses. All the data were compared between the different immunization groups by One-way ANOVA through Tukey’s post hoc tests. Kaplan–Meier curve (log-rank test) was used for tumor-bearing mice survival analyzes. Statistical significance was set at *P* < 0.05 (*: 0.05 ≥ *P* > 0.01, **: 0.01 ≥ *P* > 0.001, ***: *P* < 0.001, ns: not significant).

### Ethics statement

All experiments were done according to the Guidelines for the Care and Use of Laboratory Animals of Arak University of Medical Sciences, which refer to the American Association for Laboratory Animals Science and the guidelines laid down by the NIH (NIH Guide for the Care and Use of Laboratory Animals). All experimental protocols were approved by the ACECR (Academic Center for Education, Culture and Research) Research Ethics Committee of Mashhad, Iran (IR.ACECR.JDM.REC.1399.002).

## Results

### Production, purification, and confirmation of the recombinant multiepitope peptide vaccine

Several white colonies were screened by digestion and colony PCR to confirm the presence of the recombinant bacterial after transfection by gel electrophoresis (Fig. S2A). Also, SDS-PAGE and Western blot confirmed the recombinant multiepitope peptide vaccine production from the recombinant eukaryotic and prokaryote hosts (Fig. S2B and C). The recombinant multiepitope peptide vaccine was located near the 47 kDa band. Cation exchange and affinity chromatography methods were employed for purifying the His-tagged recombinant peptide vaccine. *E. coli* lysate solution was used as the source of the recombinant multiepitope peptide vaccine. This solution was applied to DEAE Sepharose in a low ionic strength buffer. Subsequently, the peptide vaccine was eluted from the column with the same buffer containing 1 M NaCl. The peptide vaccine appeared at ~ 5–20% salt (Fig. S3, elution time: between 176 and 207 min). Further purification of the peptide vaccine was achieved by affinity chromatography using a Ni–NTA column, and elution with buffer containing 500 mM imidazole. This two-step procedure caused purification of the His-tagged recombinant multiepitope peptide vaccine which its purity was demonstrated using SDS-PAGE (Fig. S2D). The LPS content in the purified recombinant multiepitope peptide vaccine was ≤ 9 EU/mg which is in the acceptable range^20,21^. The final yield of the recombinant peptide vaccine was 2.5 mg/mL in a volume of 20 mL which was calculated according to the Bradford method (Fig. S4).

### Humoral immune response

**Figure 2:**
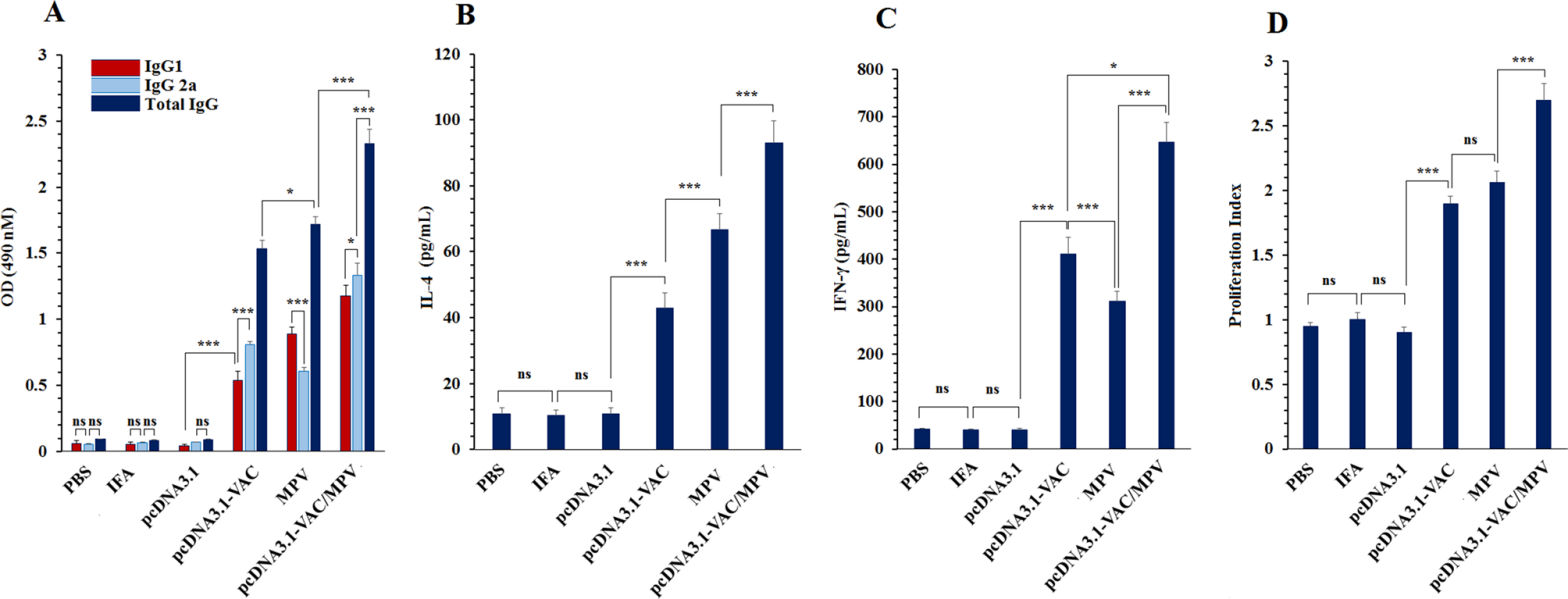
The immune response evaluation according to the serum level of antibody, cytokines secretion, and lymphocytes proliferation assay. (**A**) Serum level of the MPV-specific IgG total, IgG1, and IgG2a isotypes at different groups 2 weeks after the last injection (n = 5). (**B**) IL-4 secretion, (**C**) IFN-γ secretion, and (**D**) lymphoproliferative responses from the immunized mice splenocytes after in vitro restimulation with the MPV. 05 (*: 0.05 ≥ *P* > 0.01, **: 0.01 ≥ *P* > 0.001, ***: *P* < 0.001, ns: not significant).

The immunized mice were sacrificed 2 weeks after the last injection of the immunization schedule and their sera were collected to investigate the induced humoral immune responses in different groups. Serum level of total IgG and its subclasses (IgG1 and IgG2a) were significantly higher (*P* < 0.001) in the pcDNA3.1-VAC, MPV, and pcDNA3.1-VAC/MPV immunization groups compared with the PBS, pcDNA3.1, and IFN groups (Fig. 2A). While, no statistically significant difference (*P* > 0.05) was observed between the PBS, pcDNA3.1, and IFN groups. Furthermore, the pcDNA3.1-VAC/MPV immunized animals exhibited the highest serum level of IgG antibodies (*P* < 0.001). Besides, the level of total IgG of the MPV immunized mice was significantly (*P* = 0.0170) higher than the pcDNA3.1-VAC immunized mice (Fig. 2A). Moreover, levels of IgG subclasses were assayed to determine the provocation of Th1 and/or Th2 responses. As Fig. 2A illustrates, the pcDNA3.1-VAC vaccination mainly stimulated the predominance of IgG2a over IgG1 (*P* < 0.001) which suggests a shift toward the Th1 response. On the other side, the MPV induced shifting toward the Th2 response (*P* < 0.001). But their combined immunization regime caused significantly (*P* < 0.001) higher provocation of both arms (Th1 and Th2) in comparison to the pcDNA3.1-VAC and MPV immunization, with significant (*P* = 0.0183) predominance of IgG2a over IgG1 (Th1 shift).

### Cytokines production

Spleens of the vaccinated animals were harvested 2 weeks after the last injection and their splenocytes were cultured. The splenocytes of the pcDNA3.1-VAC, MPV, and pcDNA3.1-VAC/MPV immunized groups exhibited significantly (*P* < 0.0001) higher amounts of IFN-γ and IL-4 cytokines secretion than the PBS, pcDNA3.1, and IFN groups in response to in vitro stimulation with the recombinant MPV (Fig. 2B and C). The pcDNA3.1-VAC/MPV immunized mice splenocytes exhibited the highest (*P* < 0.0001) levels of IFN-γ and IL-4 production after stimulation. Although pcDNA3.1-VAC immunized animals’ splenocytes secreted significantly (*P* = 0.0170) higher amounts of IFN-γ in comparison with the MPV group, this ratio was vice versa for IL-4 secretion. This observation demonstrates the tendency of MPV and pcDNA3.1-VAC immunization effect to induce Th2 and Th1, respectively which is consistent with IgG isotypes predominance result.

### Lymphocytes proliferation response

Lymphocyte proliferative response was monitored using in vitro re-stimulation of the cultured splenocytes of different immunized groups with MPV (10 μg/mL). As Fig. 2D illustrates, the pcDNA3.1-VAC/MPV immunized mice exhibited the highest lymphocyte proliferation in comparison with other immunization regimes. Although MPV and pcDNC3.1-VAC vaccination caused higher lymphocyte proliferation than PBS, pcDNA3.1, and IFN groups, no significant (*P* > 0.05) difference was observed between these two groups.

### The anti-tumor effects of prophylactic immunization of the BALB/c mice with the vaccines

**Figure 3:**
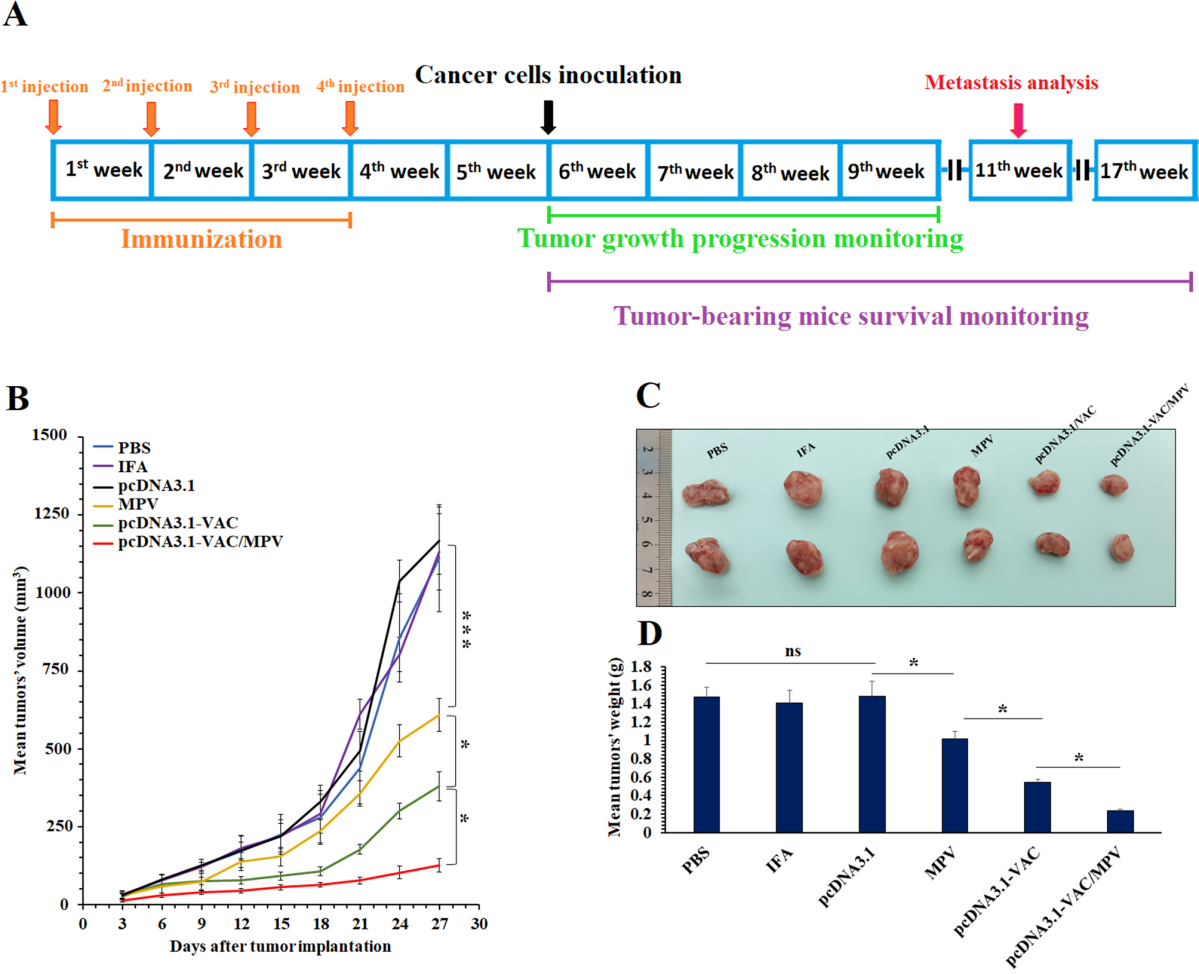
The anti-tumor effects of prophylactic immunization with the multiepitope peptide and DNA vaccines at different immunization groups. (**A**) Schematic illustration of time arrangement of immunization, cancer cells inoculation, tumor growth progression monitoring, tumor-bearing mice survival monitoring, and metastasis analysis. (**B**) The tumors’ growth progression at the PBS, IFA, pcDNA3.1, MPV. pcDNA3.1-VAC and pcDNA3.1-VAC/MPV immunized groups (n = 5). It should be mentioned that the cancer cells were inoculated two weeks after the last immunization. The tumor inoculation day was determined as day 0 at the tumor growth progression chart. (**C**) The photograph of harvested tumors on the 27th day after the cancer cells implantation (2 samples from each group). (**D**) The mean weight of harvested tumors at different immunization groups on the 27th day after the cancer cells implantation (n = 5). 05 (*: 0.05 ≥ *P* > 0.01, **: 0.01 ≥ *P* > 0.001, ***: *P* < 0.001, ns: not significant).

**Figure 4:**
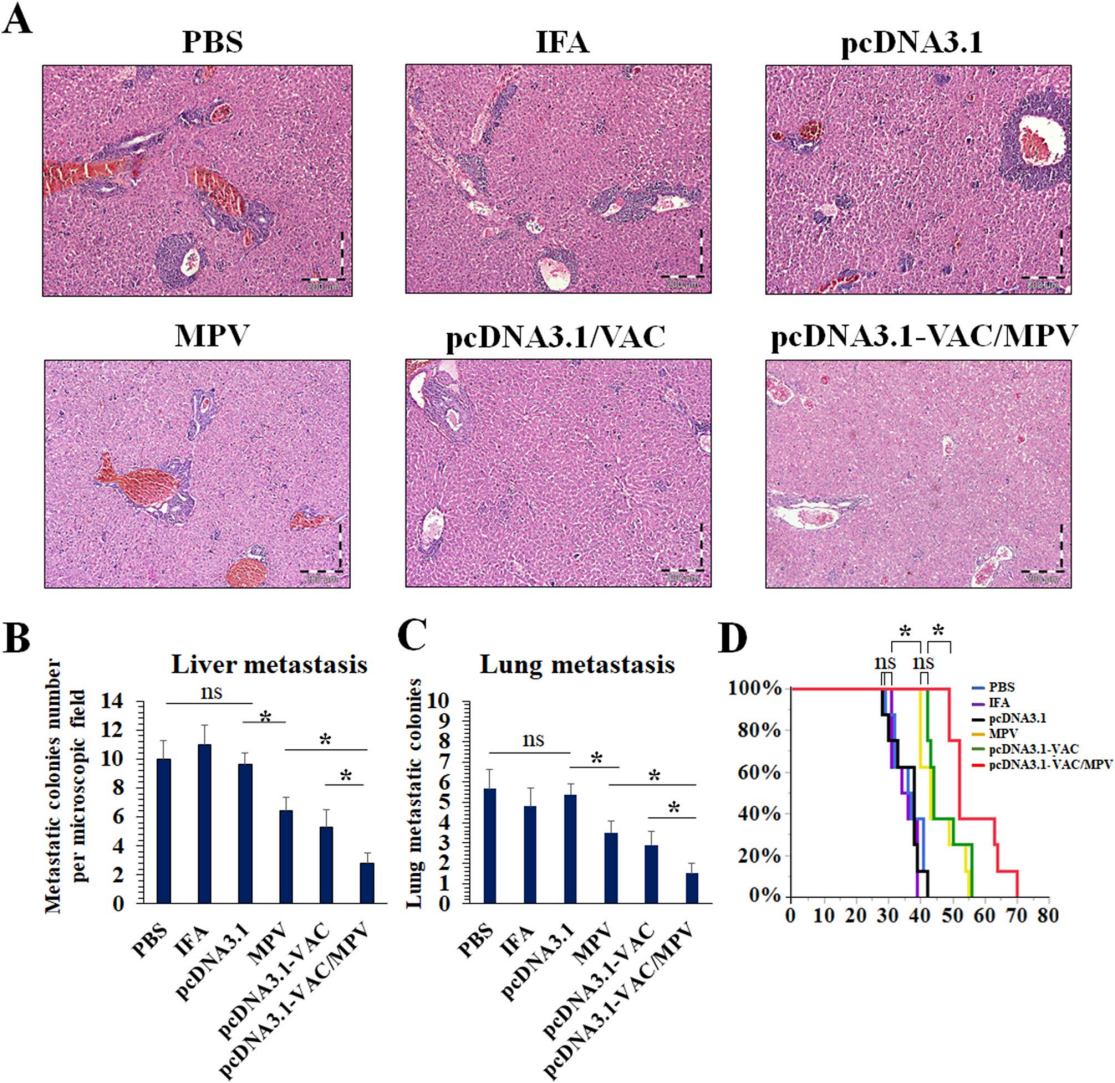
Metastasis and survival time evaluation at different immunization groups. (**A**) H&E stained sections of the immunized mice liver at 40th day after the cancer cells implantation (n = 5). (**B**) Mean metastatic colonies per microscopic field of the immunized mice at 40th day after the cancer cells implantation (n = 5). (**C**) Mean metastatic colonies of harvested lungs at 40th day after the cancer cells implantation (n = 5). (**D**) Kaplan–Meier survival curves of tumor-bearing mice at different immunization groups. (*: 0.05 ≥ *P* > 0.01, **: 0.01 ≥ *P* > 0.001, ***: *P* < 0.001, ns: not significant).

4T1 breast cancer cells were s.c injected into the mammary fat pad of the immunized BALB/c mice (cancer cells were inoculated 14 days after the last immunization (Fig. 3A)) to assess the anti-tumor effects of prophylactic immunization with the multiepitope peptide and DNA vaccines at different immunization regimes. On the last day of tumor growth progression monitoring, average tumors’ volumes (Fig. 3B and C) and weights (Fig. 3D) of the PBS, IFA, and pcDNA3.1 groups (as control groups) were almost the same. But a significant decrease was observed in the tumors’ growth progression of the MPV and pcDNA3.1-VAC immunized groups in comparison with the PBS, IFA, and pcDNA3.1 groups. The smallest mean tumors volumes and weights among all groups were observed at the pcDNA3.1-VAC/Peptide immunized group (Fig. 3B, C, and D). On the 40th day after cancer cell implantation, the liver and lungs of mice (n = 5) were harvested for analyzing metastasis burden (Fig. 4). Although MPV and pcDNA3.1-VAC exhibited a significant inhibitory effect on the metastatic colonies’ formation at these vital organs, the lowest number of metastatic colonies were detected at the pcDNA3.1-VAC/MPV immunized mice liver (Fig. 4A and B) and lungs (Fig. 4 C). Besides, the immunized mice with the pcDNA3.1-VAC/MPV (n = 8) regime exhibited the longest mean survival time among all groups (Fig. 4D). The PBS, IFA, and pcDNA3.1 groups didn’t exhibit any statistically significant difference between their mean survival times.

### Vaccine’s safety

**Figure 5:**
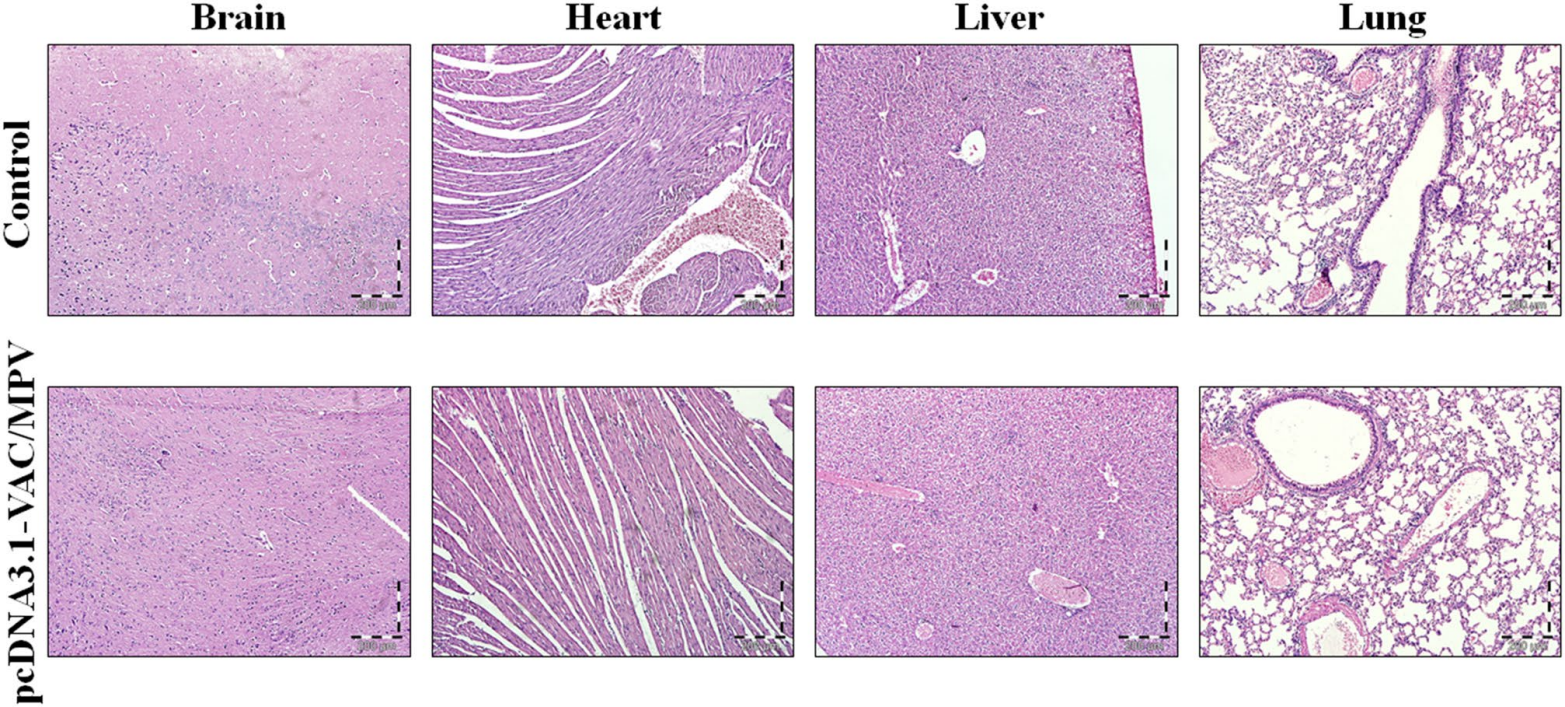
The H&E stained sections of the vital organs (brain, heart, lungs, liver) of the pcDNA3.1-VAC/MPV immunized non-tumor bearing BALB/c mice at the 30th day after the last injection.

One of the most common concerns about cancer vaccines is their safety. So, the safety of the most effective vaccination regime according to evoking immune response and anti-tumor effects was investigated. For this purpose, Non-tumor-bearing mice that were immunized with pcDNA3.1-VAC/MPV regime underwent daily monitoring for general appearance, behavioral parameters, and body weight for 30 days after the last injection. No signs of changes in the mice's appearance, behavioral pattern, food intake, and weight loss were observed during these 30 days. On the last day, the mice were sacrificed and their vital organs including the brain, heart, lungs, and liver were analyzes by histopathological exams. As Fig. 5 illustrates, no sign of inflammation, necrosis, fibrosis or any organ damage was observed in H&E-stained sections of the pcDNA3.1-VAC/MPV group in comparison with the control.

## Discussion

One of the most common target antigens for DNA and protein cancer vaccines are CTAs which have been widely investigated in numerous clinical trials^28^. Most of these trials have focused on MAGE-A CTA in whole or fusion protein forms as a cancer vaccine. For instance, from 2006 to 2016 about 44 clinical trials were initiated which used MAGE-A as the target antigen. However, the observations from their 15 largest studies (phase II or III) didn’t support considerable clinical effectiveness^29–37^. So, some concerns were raised about low immunogenicity and poor tumor-specificity of CTAs proteins immunization. To address these limitations, two popular strategies have been reported. The first is to explore new CTAs with higher immunogenicity and specificity. The second is selecting CTAs peptides consisted of the most immunodominant regions of target CTA instead of using its whole protein for optimizing their immune response^38,39^. This can be related to the complexity of the antigens' processing and presentation. The whole protein will turn to numerous numbers of products after entering the protease cleavage process and all of them should compete with each other for binding in the pockets of MHC molecules. Therefore, only a limited fraction of the MHC-presented peptides will have the chance to activate the immune response. Therefore, immunodominant epitopes of CTAs will have a better chance to be the target of the immune response when are incorporated in a peptide vaccine construct rather than the whole protein^39,40^. Immunoinformatic analyzes are the most suitable strategies to select the most immunodominant epitopes of an antigen^41–43^. Previous studies have reported that delivery of a zinc finger deleted BORIS antigen by adenoviral vectors^44^, DNA plasmid vectors^45,46^, or dendritic cell-based vaccines can activate the significant cell-mediated immune response and subsequently, anti-tumor therapeutic effects^47^. In this study, we put one step beyond and used a bioinformatics designed vaccine which contained the most immunodominant regions of BORIS antigen and multiple internal adjuvants for anti-cancer vaccination in vivo. During designing the vaccine in our previous study^17^, only completely similar regions with 100% homology between murine and human species were used. This can make the preclinical results more appliable to humans and clinics as preclinical experiments in animal models, especially murine tumor models are necessary and prerequisite before planning human clinical trials^48,49^.

Three regimes of immunization including MPV, DNA vaccine, and their combined vaccination was used in this study. To determine the predominance of Th1 or Th2 response in each immunization regime, cytokines production (IL-4 and IFN-γ) and IgG isotypes (IgG1a and IgG2) patterns were investigated. CD4 + T cells have two main subgroups including Th1 cells which secret IL-2 and IFN-γ, and Th2 subtype which secrets IL-4,5,6, and 10^50,51^. Activation of Th1 following vaccination can trigger and maintain the cellular immune response, through the specific cytokines profile secretion. On the other side, Th2 activation provokes a humoral immune response. In murine models, IgG1 subclass predominance is indicative of a Th2 response whilst the IgG2a subclass indicates more of a Th1 profile^52–54^. Taking together, cytokines production pattern and IgG isotypes distribution are indicative of immune response type. According to the cytokines and IgG isotypes production patterns in this study, the DNA vaccine caused Th1 dominant response, while MPV immunization induced Th2 dominant response. BORIS antigen as an intracellular antigen is processed for MHC-I presentation in the malignant cells which can directly activate CD8 + CTLs. This fact exhibits the determinative role of cellular immunity against BORIS-expressing tumor cells. Therefore, an ideal cancer vaccine with a BORIS target should significantly activate the cell-mediated immune response. This fact can explain the more efficient anti-tumor effects of the pcDNA3.1-VAC (with Th2 predominance) in comparison with the MPV. On the other side, combined immunization in the pcDNA3.1-VAC/MPV caused the strongest immune response in both humoral and cellular arms. Therefore, a combined immunization regime can significantly improve the MPV and pcDNA3.1-VAC efficacy. This was consistent with a significant decrease in the tumors’ growth progression, a decrease in mean tumors’ weight, a decrease in metastatic colony formation in the vital organs, and a prolongation of the tumor-bearing mice survival time. Besides, no side effects including behavioral change, weight loss, or vital organ damage according to histopathology analyzes were observed in the pcDNA3.1-VAC/MPV immunized group.

Recently, prophylactic cancer vaccines have gained lots of attention for preventing cancer development, especially in healthy persons who have high risk of developing breast cancer because of hereditary mutations in the known oncogenes or tumor suppressor genes^55–58^. On the other hand, cancer vaccines can significantly benefit patients suffering from breast cancer through inhibition of tumor recurrence or metastasis. Currently, the main therapeutic approach for most breast cancer patients is surgical resection of the primary tumor. However, tumor relapse and metastasis can be observed in a high percentage of these patients within 5 years^59^. Also, post-operation immune system suppression can considerably accelerate tumor recurrence and metastasis^60–63^. However, postoperative immune suppression is completely reversible. On the other hand, many researchers believe the failure of cancer vaccines can mostly be attributed to the negative regulatory effects of the primary tumor on the immune system. Therefore, resection of primary tumor opens a golden time window for efficient activating of the immune system against the residual cancer cells as the primary tumor’s negative regulatory effects have been omitted. Therefore, the combination of surgery and immunotherapy to prevent tumor recurrence and metastasis has gained lots of attention^64,65^. As most breast cancer patients undergo surgical resection of the primary tumor, we suggest the application of this cancer vaccine at the postoperative time window for activating the immune system against residual cancer cells and preventing tumor recurrence and metastasis.

All of this immunoprotective effect was observed in a non-immunogenic murine model. The use of immunogenic cancer cell lines may induce systemic immunity per se (without any intervention), while this phenomenon is absent in the 4T1 model as a non-immunogenic model^66^. Also, 4T1 mammary carcinoma is a highly tumorigenic, invasive, and metastatic model which has several similarities with human breast cancer^67^. However, big animals’ and humanized mice cancer models are necessary for further assessment of these vaccines’ efficacy from a different point of view as a prerequisite step for further clinical trials.

## Conclusions

In this study, we tried to produce and purify an in silico designed multiepitope cancer vaccine. This vaccine was designed by bioinformatic tools in our previous study and targets BORIS CTA. The prophylactic immunization of BALB/c mice with this vaccine caused a significant decrease in the tumors’ growth progression, inhibition of metastasis formation, and increase of tumor-bearing mice survival time compared with control groups without any side effect. These anti-cancer effects were more significant when the animals were immunized by both the DNA and peptide forms of the vaccine. Although the designed multiepitope vaccines can cause significant anti-tumor effects against murine breast cancer after prophylactic immunization, more preclinical experiments are needed to demonstrate their efficacy in different aspects.

## Supplementary Information

Supplementary Information.

## Data Availability

The data that support the findings of this study are available from the corresponding author upon reasonable request.
