# Supplementary material for: Immunoprotective effect of an in silico designed multiepitope cancer vaccine with BORIS cancer-testis antigen target in a murine mammary carcinoma model
Source: Sci Rep. 2021 Nov 30;11:23121. doi: 10.1038/s41598-021-01770-w (PMC8632969; doi:10.1038/s41598-021-01770-w)
Supplement: Supplementary file 1 — Supplementary Information. [file 41598_2021_1770_MOESM1_ESM.docx]

**Immunoprotective effect of an *in silico* designed multiepitope cancer vaccine with BORIS cancer-testis antigen target in a murine mammary carcinoma model**

*Elham Mahdevar* ^1^*, Amirhosein Kefayat* ^2^*, Ashkan Safavi* ^3*^, *Amirhosein Behnia ^4^, Seyed Hossein Hejazi ^5^, Amaneh Javid* ^1^, *Fatemeh Ghahremani ^6*^*

^1^ Department of Biology, Faculty of Science and Engineering, Science and Arts University, Yazd, Iran.

^2^ Department of Oncology, Cancer Prevention Research Center, Isfahan University of Medical Sciences, Isfahan, Iran.

^3^ Department of Biology, Science and Research Branch, Islamic Azad University, Tehran, Iran.

^4^ Department of Biology, Faculty of the Basic Sciences, Shahrekord Branch, Islamic Azad University, Shahrekord, Iran.

^5^ Department of Parasitology and Mycology, School of Medicine, Isfahan University of

Medical Sciences, Isfahan, Iran.

^6^ Department of Medical Physics and Radiotherapy, School of Paramedicine, Arak University of Medical Sciences, Arak, Iran.

**Corresponding authors ***:

- **Ashkan Safavi**

Department of Biology, Science and Research Branch, Islamic Azad University, Tehran, Iran. Mobile: +98 9123196231

Email: ashkan.safavy@gmail.com

- **Fatemeh Ghahremani**

Department of Medical Physics and Radiotherapy, School of Paramedicine, Arak University of Medical Sciences, Arak, Iran.
Mobile: +98 9183162173

Email: F.ghahremani@arakmu.ac.ir

**Running title:** Multiepitope DNA and peptide cancer vaccines targeting BORIS


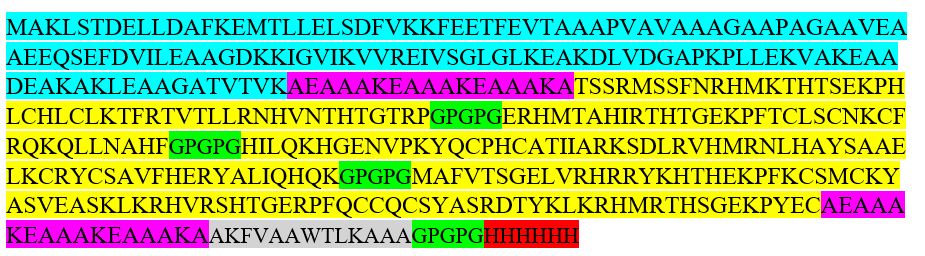


**Figure S1:** The amino acid sequence of the final vaccine construct (431 amino acids). The vaccine construct has three domains including L7/L12 ribosomal protein (blue highlights), the four selected immunodominant regions of the BORIS antigen (yellow highlights) including the 265-312, 320-400, 416-478, and 498-533 residues of BORIS antigen which are merged by GPGPG linkers, and the PADRE sequence as a universal T helper epitope (gray highlights). These domains were merged together by GPGPG (green highlights) and A(EAAAK)3A linkers (pink highlights). His-tag sequence at the C-terminal of the vaccine is indicted by red highlights.


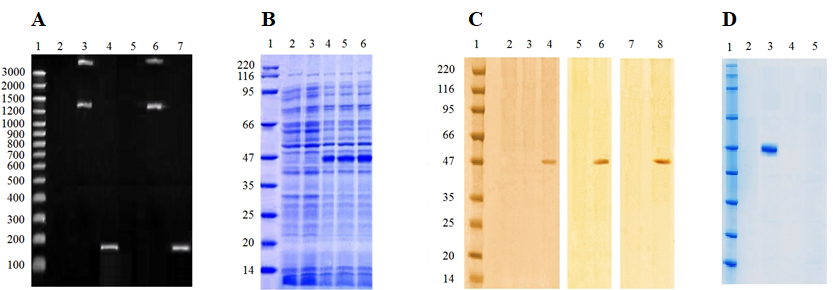


**Figure S2**: Transfection efficacy monitoring by electrophoresis gel and detection of the recombinant multiepitope peptide vaccine production by SDS-PAGE and Western blot. (A) Monitoring of the prokaryotic host transfection by gel electrophoreses (lane 1: DNA ladder 100-3000 bps, lane 2: PBS as the negative control, lane 3: Digestion of the extracted pcDNA3.1-VAC by Nco1 and Xho1 enzymes, lane 4: The colony PCR evaluation of the pcDNA3.1-VAC transfected bacteria, lane 5: Negative control, lane 6: Digestion of the extracted pET-28a-VAC by Nco1 and Xho1 enzymes, lane 7: The colony PCR evaluation of the pET-28a-VAC transfected bacteria). (B) The recombinant multiepitope peptide vaccine expression analysis by SDS-PAGE (lane 1: Protein ladder 14-220 kDa, lane 2: The cell lysate of non-transfected *E. coli* BL21 as the negative control, lane 3: The cell lysate of *E. coli* BL21 transfected by empty pET-28a vector, lanes 4-6: The cell lysate of *E. coli* BL21 transfected by pET-28a-VAC in 0.5, 0.75, and 1 mM IPTG concentrations). (C) The recombinant multiepitope peptide vaccine expression monitoring by Western blot (lane 1: Protein ladder 14-220 kDa, lane 2: untransformed L929 cell lysate. lane 3: The lysate of transfected L929 cells by empty pcDNA3.1. lane 4: The lysate of transfected L929 cells by pcDNA3.1-VAC. lane 5: The cell lysate of untransformed *E. coli* BL21. lane 6: The cell lysate of transfected *E. coli* BL21 by pET-28a-VAC. lane 7: PBS as the negative control. lane 8: The purified recombinant multiepitope peptide vaccine by Ni-NTA column). (D) The purity of the recombinant multiepitope peptide vaccine was evaluated by SDS-PAGE (lane 1: Protein ladder 14-220 kDa, lane 2: PBS as the negative control, lane 3: The purified recombinant multiepitope peptide vaccine by Ni-NTA column).


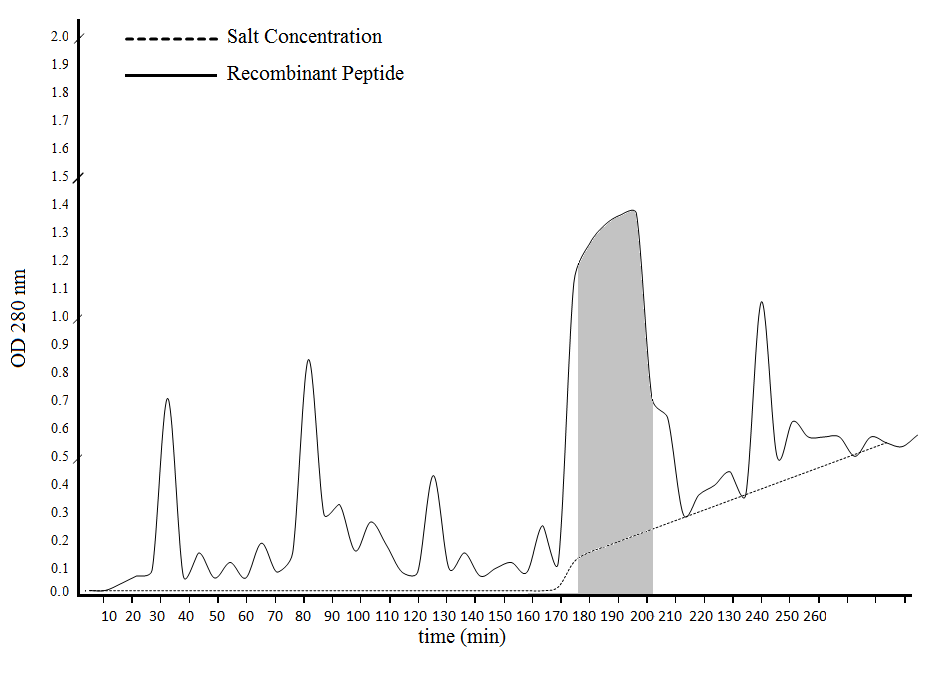


**Figure S3:** Purification of the His-tagged recombinant multiepitope peptide vaccine using DEAE Sepharose. The gray area represents pooled fractions of the peptide vaccine. Pooled fractions were dialyzed and purified further using the Ni-NTA column.


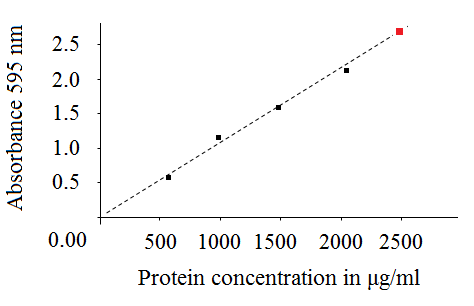


**Figure S4:** Analysis of the purified recombinant peptide vaccine by Bradford method.
